# Responsiveness of hand-held dynamometry for measuring changes in trunk muscle strength in people with chronic low back pain

**DOI:** 10.1186/s12891-025-08325-4

**Published:** 2025-01-18

**Authors:** Shouq Althobaiti, Janet A. Deane, Deborah Falla

**Affiliations:** 1 Centre of Precision Rehabilitation for Spinal Pain (CPR Spine), School of Sport, Exercise and Rehabilitation Sciences, College of Life and Environmental Sciences, University of Birmingham, Birmingham, B15 2TT UK; 2 Physical Therapy Department, College of Applied Medical Science, Taif University, Taif, Saudi Arabia

**Keywords:** Hand-held dynamometer, Responsiveness, Trunk isometric strength, Chronic low back pain, Performance-based outcome measures

## Abstract

**Objective:**

To assess the responsiveness of a hand-held dynamometer (HHD) in evaluating changes in trunk isometric strength in people with chronic low back pain (LBP).

**Background:**

Reduced trunk muscle strength has been associated with pain incidence and severity in people with chronic LBP. Trunk muscle strength is an important functional outcome that is measured in clinical practice and research. However, the responsiveness of clinical tools such as HHD for measuring changes in trunk muscle strength remains underexplored.

**Methods:**

Maximum isometric trunk strength was measured using both a HHD and an isokinetic dynamometer (ID) in 21 participants with chronic LBP both before and after 6 weeks of progressive trunk resistance exercises. Effect sizes (ES) and standardised response mean (SRM) were used to evaluate the internal responsiveness of the HHD measures. External responsiveness was determined by correlating the change scores measured with the HHD with those obtained using the ID.

**Results:**

Following the progressive resistance exercise programme, there was a significant improvement in trunk muscle strength measured with the HHD with moderate to large ES (0.40–0.85) and SRM (0.60- 0.74), indicating moderate to high internal responsiveness. Pearson's correlations revealed a weak correlation between changes in trunk strength measured with the HHD and those measured with the ID (*r* = 0.22- 0.26), indicating inadequate external responsiveness.

**Conclusions:**

Although the use of a HHD was shown to have internal responsiveness for detecting changes in trunk muscle strength, the inadequate external responsiveness warrants further investigation. Future research should also explore the responsiveness of HHD in people with chronic LBP with higher pain and disability levels using comparable measurement setups.

**Supplementary Information:**

The online version contains supplementary material available at 10.1186/s12891-025-08325-4.

## Introduction

With a point prevalence of 540 million, low back pain (LBP) is a highly prevalent musculoskeletal condition and the leading cause of years lived with disability globally [1]. People with chronic LBP commonly exhibit multiple biophysical, social, and psychological features that subsequently affect their functional status and quality of life [1].

When considering physical features in people with chronic LBP, numerous studies have documented reduced trunk strength and endurance and altered activity of trunk muscles in people with chronic LBP [2–8]. Additionally, trunk muscle weakness has been associated with LBP incidence and severity [9]. Given the relevance of adequate trunk muscle strength for people with LBP [10, 11], accurate evaluation of an individual's trunk strength using objective tools is important for both clinical and research evaluative, discriminative, and predictive purposes [12, 13]. This implies that measures of trunk strength must have suitable psychometric properties for their intended use [14].

Multiple performance‐based outcome measures (PBOMs) have been utilized to evaluate trunk muscle strength [15–17]. The isokinetic dynamometer (ID) is the gold standard [18] and is used to measure trunk muscle strength across various angular velocities and contraction modes (i.e., isometric, concentric and eccentric), allowing the calculation of agonist/antagonist ratios [19]. In terms of psychometric properties, IDs are valid [20], reliable [21], and sufficiently responsive to identify muscle weakness [22] and improvements in muscle strength following interventions for individuals with chronic LBP [23, 24].

A hand-held dynamometer (HHD) is an objective, portable and clinically applicable measurement instrument that can also be used to evaluate trunk muscle strength [15, 25]. Recently, the reliability and criterion validity of the HHD has been determined in people with chronic LBP, demonstrating good to excellent within-day and between-day reliability with high intraclass correlation coefficients (ICC _(2,1)_) of 0.73–0.93 and moderate to strong correlations (*r* = 0.68–0.78) between HHD and Biodex ID strength measurements [26]. However, to date, the responsiveness of HHD has not been assessed for the measurement of trunk muscle strength [27].

According to COnsensus-based Standards for the Selection of Health Measurement INstruments (COSMIN), responsiveness is defined as the ability of a measure to detect change over time in the construct of interest [28]. Evaluating whether a person's muscle strength has changed over time is often the primary goal in clinical practice and research [14]. The present study aimed to assess both the internal and external responsiveness of HHD for trunk muscle strength assessment in people with chronic LBP following 6 weeks of progressive resistance exercise of the trunk.

## Methods

### Study design

This study used a prospective cohort design with a follow-up at 6 weeks. Twenty-two individuals with chronic nonspecific LBP were recruited from the University of Birmingham student and staff population. The sample size was calculated using G*Power software based on a 0.05 significance level, power of 80% and an expected correlation of 0.6 between measures with the HHD and the ID. This calculation indicated a need for 19 participants. To account for possible dropouts, a total of 22 participants were recruited.

The study was conducted at laboratories within the Centre of Precision Rehabilitation for Spinal Pain (CPR Spine), University of Birmingham, UK from September 2022 to July 2023 following the Declaration of Helsinki. It was approved by the University of Birmingham ethics committee (ERN_22-0512). All participants signed an informed consent form prior to data collection.

### Participant eligibility criteria

We recruited people aged 18 to 55 years who had chronic nonspecific LBP (lasting at least three months over the past six months) [29], with a pain rating of 2 or higher on a 0–10 numerical pain rating scale (NPRS) and moderate disability of at least 20% on the Oswestry Disability Index (ODI) [30]. Due to the nature of the measurement tasks with repeated strength testing, we limited the maximum age to 55 years. Those with signs of serious trauma or pathology, cardiovascular diseases, cancer, fractures, spinal stenosis, systemic or inflammatory conditions, neuromuscular disorders, pregnancy, current active low back pain management, participation in competitive athletics, or engaging in over 90 min of vigorous exercise daily were excluded from the study.

### Patient-reported outcome measures

Several patient-reported outcome measures (PROMs) were administered at baseline and follow-up, all of which have adequate psychometric properties for people with chronic LBP. These measures included LBP intensity (NPRS) [31, 32], perceived disability (ODI) [33], overall health status using the 36-Item Short Form Survey (SF-36) [34], physical health via the International Physical Activity Questionnaire (IPAQ) [35], and measurement of fear of movement using the Tampa Scale for Kinesiophobia (TSK) [36].

### Dynamometry

**Fig. 1:**
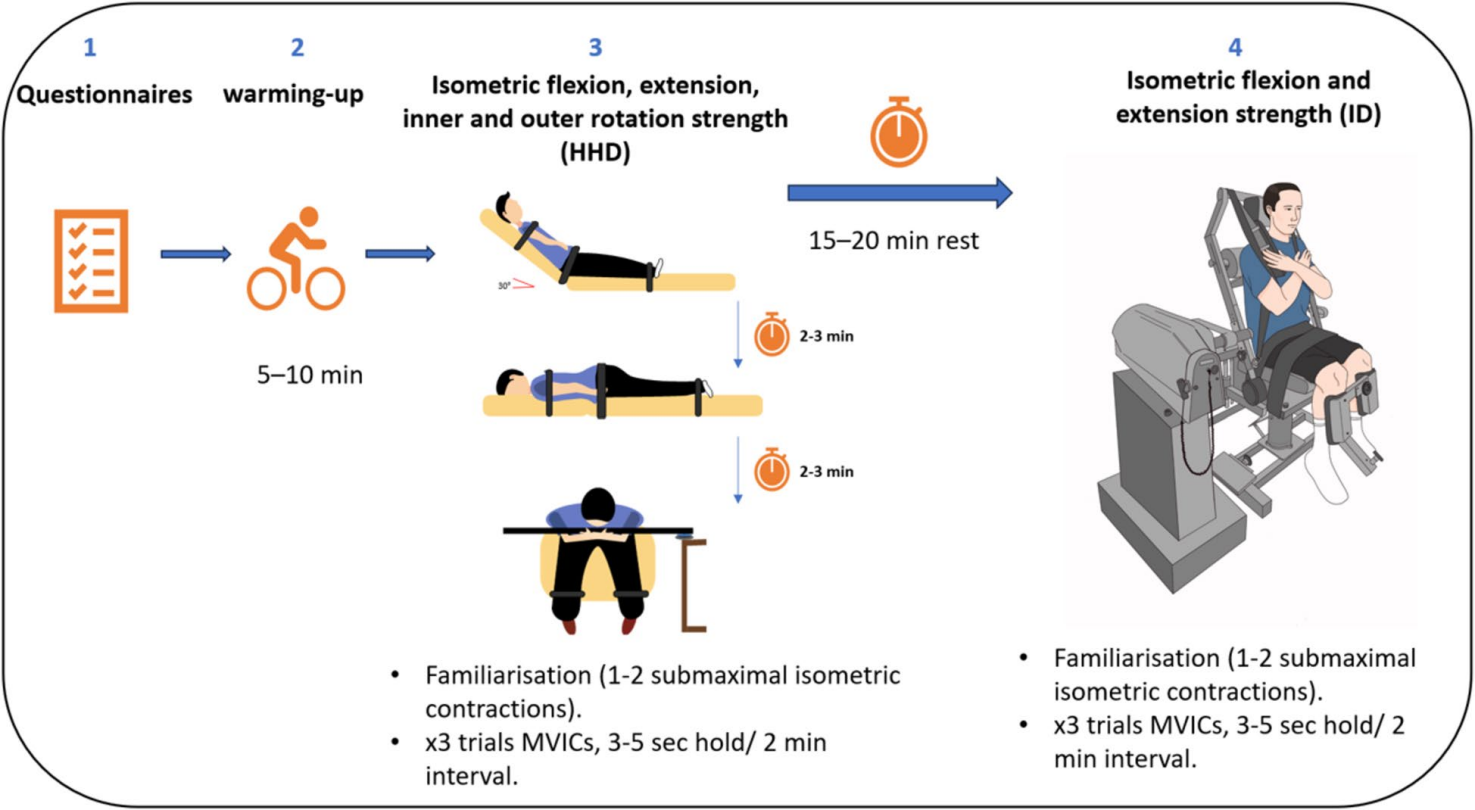
Overview of the experimental setup conducted for both baseline and follow-up evaluation sessions

Trunk maximum voluntary isometric contraction (MVIC) was measured using two different dynamometers, an Active force 2 HHD (Active body Inc., USA) and an ID (System 3 Pro, Biodex Medical Systems, USA), both at the beginning and immediately after the six-week training period. For the HHD measurements, the participant was comfortably positioned into the testing positions as illustrated in Fig. 1, with stabilisation around the pelvic and ankles and the HHD was secured with a strap just under the suprasternal notch for flexion (from 30° supported supine), and at the level of T4 for extension strength measurements (from a prone position). Rotation strength was measured from sitting with the hips and knees flexed to 90° and feet off the floor, with a belt secured around the pelvis and proximal to the knees to limit the engagement of lower extremities. While holding the bar with an upright position, participants were directed to rotate their trunk and push against the HHD, on the most painful side. Participants were instructed to push forward to measure inner rotation strength and to push backward for outer rotation strength [26]. The back extension-flexion attachment was used for the MVICs performed on the ID where participants maintained a seated compressed isolated lumbar position with their hips and knees positioned at 90° of flexion, feet parallel to the floor and spaced shoulder-width apart. The dynamometer was aligned with the front of the seat tilted approximately 15° clockwise. To minimise compensatory movements, straps were secured around the participants' thighs, pelvis, and upper trunk, and a specific attachment was used to restrict knee movement. The dynamometer was locked to maintain a hip-to-trunk angle of 90°. This position was achieved by locking the dynamometer at 50° based on its goniometer as recommended by the manufacturer [37, 38].

### Experimental procedure

Following completion of the questionnaires, the participants performed a 5–10-min warm-up on a stationary bicycle. They were then introduced to the procedures of measuring trunk strength using both the HHD and the ID [39]. The overall procedure is illustrated in Fig. 1. Standardized verbal instructions were given to the participants on how to perform the tasks, and they were verbally encouraged to exert their maximum effort during MVIC testing. Participants were given breaks between trials and tasks to allow for recovery [40].

To maintain consistency, all measurement sessions were conducted at the same time of day to minimize diurnal variations [41] and by the same physiotherapist (with 2 years of experience and training with the testing equipment). The tasks in all sessions were performed in a semi-random order: HHD was always followed by ID, but a random order of measurements for each dynamometer was determined using a random number generator. The same sequence was retained for the post-training evaluation session, which took place 3–7 days following the final exercise session. Both the HHD and the ID were calibrated before the evaluation took place.

### Progressive resistance exercise

**Fig. 2:**
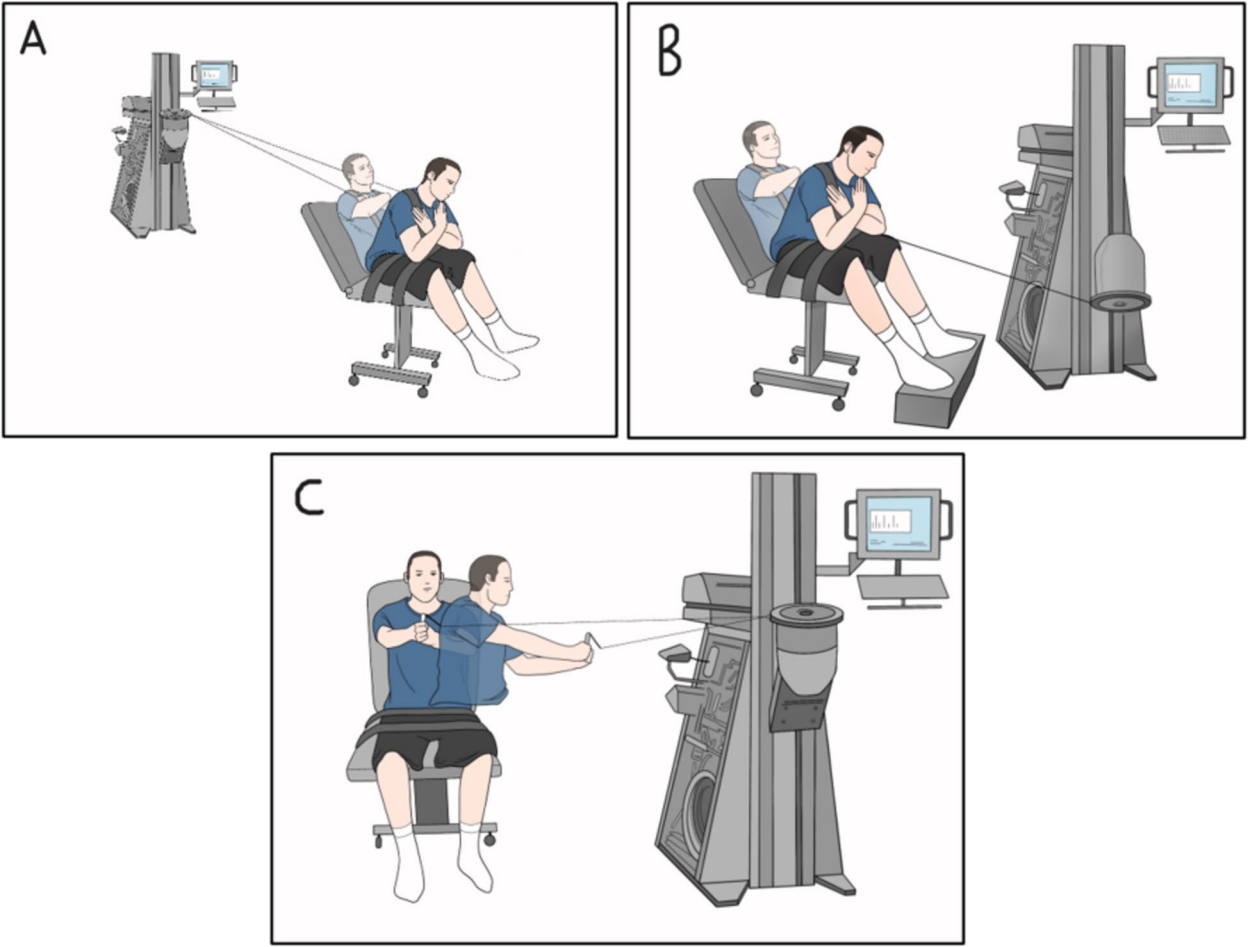
Illustration of the progressive resistance exercises of trunk. **A** flexion, (**B**) extension and (**C**) rotation using the Primus functional dynamometer (BTE Technologies, USA)

A functional dynamometer (Primus, BTE Technologies, USA) was used to perform progressive isotonic resistance exercises targeting the trunk muscles. These exercises included trunk flexion, extension, and right and left rotation and were performed in random order while seated on the Primus chair, with straps secured around the thighs and pelvis to minimize their involvement [42, 43]., Fig. 2 illustrates the exercise setup.

Before commencing the training program, participants underwent three MVIC trials for each movement in the mid-position to determine the appropriate training load for each exercise. The training load was tailored to each participant’s individual maximum effort (50%-80% of MVIC) and adjusted weekly based on their performance and capacity.

The six-week training programme consisted of supervised progressive resistance exercises conducted at the laboratory once per week, with each session lasting approximately one hour [44]. Participants were supervised by the same assessor, who provided standardised verbal instructions and feedback to ensure the correct technique and consistency across sessions. This was done through monitoring the Primus exercise head position and angle, seat hight and participant position and movement. Each session began with a 5–10-min general warm-up on bicycle, followed by tailored warm-up exercises specific to the four movement being trained. These included one set of 10 repetitions at 50% of the training load [45], designed to prepare the participants for the subsequent resistance exercises.

The main training sets consisted of two sets of isotonic (concentric/eccentric) resistance exercises performed at 50%-80% of MVIC, with 8–15 repetitions per set and one-minute rest intervals between sets, as recommended for people with chronic LBP [46, 47]. Load progression was implemented by increasing the resistance by by 5% in each session provided participants successfully completed their exercises without significant difficulty or discomfort. Progression was also tailored to accommodate individual limitations, such as pain thresholds or fatigue levels, ensuring the program remained both challenging and safe. Participants were instructed to perform concentric movements over two seconds, hold for approximately one second, and complete eccentric movements over four seconds [48].

Each session concluded with a 10-min cool-down period, which included standardised mobility and static stretching exercises for the back and abdominal muscles. Specific stretches included movements such as knee hugs and child’s pose, held for 15–30 s each. Additionally, mobility exercises such as knee rolls, camel and cat movements were performed 5–10 times. Participants were advised not to perform additional trunk-specific exercises outside the supervised training sessions to standardise the intervention and reduce variability.

Adherence to the program was monitored by recording attendance at the weekly training sessions. Participants who missed a session were offered a make-up session within the same week to maintain consistency in the intervention.

### Statistical analysis

Peak strength (highest value among the three MVICs) from the HHD was recorded in Newtons, and the highest peak torque exerted during the MVICs performed on the ID was recorded in Newtons/meter. All statistical analyses were conducted using a statistical software package (IBM SPSS version 28, USA) assuming a 0.05 significance level. Descriptive statistics, represented as the mean ± standard deviation (SD), were used to describe the participants’ characteristics and the outcome variables. Initially, the normality of the data was confirmed using the Shapiro–Wilk test. A paired sample t-test was then used to test for the differences in the outcome measures before and after the exercise program. Effect size (ES) mean change scores/SD of baseline scores [49] and the standardised response means (SRMs) were computed as the change scores divided by the SD of change scores [50] to evaluate the magnitude of the experimental effect and to assess the internal responsiveness of the HHD measures. Internal responsiveness is defined as the ability of the outcome to measure change over time [51, 52]. Cohen d was used to interpret the magnitude of the ES and the SRMs as 0.20–0.49 small, 0.50–0.79 moderate and ≥ 0.80 indicating a large ES [49].

As per COSMIN guidelines, external responsiveness (criterion responsiveness) was calculated using the correlation of changed scores between HHD and criterion gold standard ID measures [14]. We examined correlations between the HHD and ID measures for both flexion and extension. The criterion responsiveness refers to the degree to which the change scores of the outcome of interest are an adequate reflection of the change scores of the ‘gold standard’ [53]. Based on the data normality, either Pearson product-moment or Spearman rank correlation coefficient* r* was used to explore the criterion responsiveness of the HHD. The *r* values of 0.00—0.39, 40–0.69 and > 0.70 were interpreted as weak, moderate, and strong correlations, respectively [54]. However, as per COSMIN guidelines, a correlation or Area Under the Curve (AUC) of at least ≥ 0.70 is usually considered to indicate adequate responsiveness [53].

## Results

Out of the initial 22 participants enrolled in the study, one individual withdrew due to an increase in LBP, which according to the participant, was unrelated to the measurements or the training protocol. Consequently, the data from 21 participants were used for both baseline and follow-up evaluations. Participants’ characteristics are presented in Table 1 as the mean and standard deviation (SD).

**Table 1:** Participants’ characteristics

Characteristic	Mean (SD)
Age (years)	33 (7.2)
Gender (females %)	61%
Weight (kg)	72. (13.6)
Height (cm)	169.6 (10.7)
BMI (kg/cm^2^)	24.9 (3.7)

*Kg* kilograms, *cm* centimetres, *BMI* body mass index

All PROMs and PBOMs showed improvement at 6 weeks compared to baseline scores, and most of the outcome measures showed a statistically significant change (Table 2).

**Table 2:** Patient-reported and performance‐based outcome measures at baseline and follow-up

Characteristic	Mean (SD)		*p*-value
	**Baseline**	**Follow-up**	
**Pain intensity (NPRS)**			
Average pain intensity in the past week	4.4 (2.1)	2.1 (1.6)	< .001***
Average pain intensity in the past 24 h	3.7 (1.8)	2.5 (2.3)	0.24
Current pain intensity	3.4 (1.7)	1.7 (1.3)	.001**
**ODI %**	41.1 (8.8)	33.2 (7.5)	< .001***
**TSK**	38.3 (8.3)	35 (8.3)	.003**
**SF-36**			
Average physical functioning	62.8 (16.5)	77.8 (14.7)	< .001***
Limitation due to Physical health	55.9 (43.2)	66.6 (38.9)	.215
Limitation due to Emotional problems	46 (40.1)	84.1 (27.1)	< .001***
Energy/Fatigue	43.5 (18.3)	49.7 (17.2)	.058*
Emotional well-being	59.6 (22.6)	68.3 (18.6)	.008*
Social Functioning	64.2 (21)	75.5 (18.7)	.007*
Pain	55.9 (17.7)	66.6 (19.6)	.022*
General health	55.2 (17.2)	59 (17.7)	.186
**IPAQ**			
Walking (min)	1703.4 (1080.8)	2937 (3486.1)	.128
Moderate (min)	2406 (3261.8)	2617.76 (3373.8)	.941
Vigorous (min)	3217.7 (3794.8)	3004.4 (4520.3)	.708
**ID-Flex (N/m)**	93.8 (43)	111.2 (43.5)	.001**
**ID-Ext (N/m)**	110 (61.8)	125.8 (56.8)	.197

*NPRS* numeric pain rating scale, *ODI* Oswestry Disability Index, *IPAQ* International Physical Activity Questionnaire, *TSK* Tampa Scale for Kinesiophobia, *SF-36* 36-Item Short Form Survey, *cm* centimetres, *ID* isokinetic dynamometer, *Flex* flexion, *Ext* extension. Significant differences are marked with asterisks (*p < 0.05, **p ≤ 0.005, ***p < 0.001)

### HHD internal responsiveness

Participants' trunk muscle strength scores were significantly better at the follow-up than at baseline (*p* < 0.05), with moderate to large ES and SRM (Table 3).

**Table 3:** HHD internal responsiveness

Task (N)	Mean (SD)		Change (N)	*p*-value	ES	SRMs
	**Baseline**	**Follow-up**				
HHD-Flex	82.9 (33.8)	107 (37.7)	24.1	.010*	0.71	0.61
HHD-Ext	125.8 (45.4)	164.9 (49.5)	39	.003**	0.85	0.74
HHD-InnRot	42.5 (30.3)	61.8 (40.5)	19.2	.005**	0.63	0.67
HHD-OutRot	34.8 (28.8)	46.4 (29.4)	11.6	.012*	0.40	0.60

*ES* effect size (Cohen's d), *SRMs* standardized response means, *HHD* Hand-held dynamometer, *N* Newtons, *Flex* flexion, *Ext* extension, *InnRot* inner rotation, *OutRot* outer rotation. Significant differences are marked with asterisks (*p < 0.05, **p ≤ 0.005)

### HHD external responsiveness

According to the correlation analysis, a weak and non-statistically significant, correlation was reported between HHD and ID change scores (Table 4).

**Table 4:** External responsiveness: Correlations between HHD and ID

Task	Correlation *r*	Sig. (2-tailed)	95% confidence interval CI	
			**Lower**	**Upper**
**HHD-Flex** **ID- Flex**	0.22	0.32	-0.22	0.60
**HHD-Ext** **ID-Ext**	0.26	0.24	-0.18	0.62

HHD hand-held dynamometer, *ID* isokinetic dynamometer, *Flex* flexion, *Ext* extension.* r* = Pearson’s correlation

## Discussion

Trunk muscle strength is one of the most fundamental objective parameters for determining clinical progress and treatment effectiveness in people with chronic LBP, highlighting the importance of having a responsive, clinically applicable instrument to assess this outcome. To our knowledge, the current study is the first to evaluate the responsiveness of HHD for measuring isometric trunk muscle strength following progressive resistance exercises in people with chronic LBP.

The results of the current study indicate that HHD has moderate to large internal responsiveness (i.e., moderate to large ES and SRM). However, when considering external responsiveness (i.e., relative to the ID measures), none of the measures were responsive, with only weak correlations identified. We assessed internal responsiveness by calculating both ES and SRM, which are commonly utilized in responsiveness studies to examine the mean change and the magnitude of measured changes [52, 55]. However, it is important to note that, in accordance with COSMIN recommendations, t-tests, ES, and SRM are considered to be insufficient measures of responsiveness, as they primarily emphasize the magnitude of change scores rather than the validity of these changes [14]. Therefore, we assessed both internal and external responsiveness, which is a strength of the current study.

We identified inadequate external responsiveness of the HHD, as the correlations of HHD measures with the ID measures of trunk strength were weak and not statistically significant. A correlation of ≥ 0.70 is considered adequate for the tool to have criterion responsiveness, as per COSMIN recommendations [53]. It is well known that assessing maximum muscle strength can be influenced by a range of factors, including patient cooperation and motivation [22]. Even though best practice was followed during testing through performing familiarization trials, randomization of measured tasks, standardization of testing protocols, and instructions, it is impossible to rule out that each measurement instrument was accompanied by a certain degree of measurement error. Hence, lower correlations between changes in the instrument scores and the changes in the criterion scores should be anticipated [14]. Another reason for the inadequate external responsiveness may be related to the different testing postures used by the HHD and ID. HHD testing was conducted in a lying position (30° supported for the trunk flexion task), while ID testing was performed in a seated position with hips and knees flexed to 90°. Since trunk and hip muscles function synergistically, these positional differences could affect the levels of trunk strength measured and, consequently, the observed changes in strength scores obtained by both instruments [56]. The influence of testing posture on trunk muscle strength has been explored in the literature, with several notable findings. When lying (hips and knees at 90° during flexion and straight during extension) was compared to sitting (hips and knees at 90°), the maximal torque of trunk flexors was similar between the two positions, with no significant differences detected. However, the torque of trunk extensors was significantly greater in the sitting position compared to the lying position [57]. Similarly, another study reported that the highest average force for trunk extension was observed in the sitting position, followed by kneeling and standing [58]. These findings suggest that posture-specific biomechanical factors play a critical role in trunk muscle strength outcomes. Variations in muscle length-tension relationships relative to hip position have a relatively minor effect on the iliopsoas' contribution to trunk strength during flexion tasks, owing to its relatively short length and monoarticular nature [59]. Conversely, the optimal length-tension relationships of hip extensors in the sitting position, combined with the larger cross-sectional area of synergistic muscles such as the gluteus maximus and hamstrings, contribute to higher trunk extensor torque. Furthermore, the increased mechanical advantage of erector spinae activity in more flexed posture may selectively enhance trunk extensor strength output [60].

Differences in posture are likely to alter the distribution of compression and shear forces within the spine and may recruit deep and superficial muscles differently to stabilise the vertebrae [61]. Additionally, these variations could impact symptom provocation in individuals with chronic low back pain, potentially influencing their effort or ability to generate maximal force [62]. While this discrepancy introduces confounding variables, it also mirrors clinical reality, where different setups and stabilisation methods are routinely employed. Despite these limitations, the use of distinct postures in this study reflects the practical and diverse contexts in which HHD is applied in real-world clinical settings, adding to its validity.

It is challenging to directly compare the magnitudes of responsiveness obtained in the present study with those reported in the literature, as there is a notable lack of research investigating responsiveness in performance-based tests for individuals with chronic LBP [63]. A recent systematic review [27], found no studies evaluating the responsiveness of trunk muscle strength measures in this population. However, the responsiveness of HHD has been investigated for muscle groups and populations, such as quadriceps and neck muscles, following specific interventions [64–66]. For example, after total knee arthroplasty, quadriceps strength assessed with HHD demonstrated a high responsiveness, with an SRM exceeding 1.57 [65]. Similarly, a study measuring neck retraction strength in individuals with neck pain reported a large SRM of 1.57 N, detecting a substantial improvement from 76.5 N to 119.5 N following physical therapy [66]. These findings suggest that HHD can detect meaningful changes in muscle strength across various contexts, although further research is needed to establish its responsiveness for trunk muscle strength in individuals with chronic LBP.

The responsiveness of the number of generic and disease-specific PROMs for people with chronic LBP has been previously examined [55, 67–69]. These studies highlight the utility of PROMs in capturing clinical changes over time. In contrast, fewer studies have investigated the responsiveness of other PBOMs, such as walking tests, sit-to-stand tests, stair climbing tests and loaded forward reach tests [55, 63, 70]. The responsiveness of the progressive isoinertial lifting evaluation test (PILE) [71], which is a dynamic test to evaluate trunk strength and lifting capacity, was examined in people with chronic LBP before and after 10 weeks of physical therapy, including active physical therapy, cognitive behavioural therapy or both. The responsiveness of the PILE test measured by the AUC was 0.59 [63], which is considered inadequate [53]. Contrary to this finding, Ljungquist et al. [70] reported adequate responsiveness of the lumbar PILE test in people with spinal pain. This discrepancy might be attributed to different responsiveness criteria and the statistical tests conducted.

In accordance with the present results, previous studies have demonstrated similar improvements in isometric extension strength (+ 59.2 Nm/kg; *P* < 0.05) and significant improvement in pain and pain-related limitations following several weeks of isodynamic lumbar extension exercises [58]. Another study found a statistically significant improvement in isometric lumbar extension strength (*P* < 0.05) in people with chronic LBP who were randomly assigned to either limited or full range of lumbar extension strength training at 80% of their MVC compared to the control group [47]. Although there was an improvement in trunk extension strength measured with the ID in the current study, it is important to note that this change was not statistically significant, which is in contrast to the significant improvement in trunk extension strength measured with the HHD. It should be noted, however, that there is a distinction between these measures with trunk extension strength assessed with the HHD versus isolated lumbar extension strength measured with the ID [10].

The exercise programme utilised in the current study generally focused on enhancing trunk extension strength, in addition to flexion and rotation strength. Although best efforts were made to minimize the engagement of the pelvis and legs through adequate restraint techniques, it is evident that these measures alone were not necessarily sufficient to elicit a significant improvement in isolated lumbar extension strength. A similar finding was previously reported where no improvement in isolated lumbar torque was observed for a group who participated in trunk extension training without pelvic stabilization compared to a group that trained on a lumbar extension machine that provided pelvic stabilization [72, 73].

Collectively with the established reliability and criterion validity of the HHD [26], these findings (moderate to large ES and SRM) support the potential clinical usefulness of the HHD for monitoring changes in isometric trunk muscle strength. However, the poor correlation of strength change between the HHD, and the ID (external responsiveness) suggests that this tool might not be a reasonable and clinically feasible substitute for the ID. Furthermore, it's important to exercise caution when comparing results obtained from these tools, certainly not using the scores of both devices interchangeably. This underscores the significance of comprehending the distinctions and potential limitations of each tool in clinical assessment.

### Methodological considerations

This study has some limitations that should be considered when interpreting the results. Firstly, the current study was conducted on a sample of patients with chronic LBP, with the majority recruited from a university population. This specific demographic sample may not necessarily reflect the characteristics and health status of the broader general population with chronic LBP. The participants in this study were relatively young (mean age of 33 years), presenting with moderate pain and disability. Therefore, future studies should consider recruiting participants seeking clinical care with a higher degree of pain and disability to confirm the responsiveness of HHD when used in this population.

Secondly, the absence of randomisation in the sequence of measurements (HHD versus ID) could be a potential limitation. In this study, HHD measurements were consistently performed before ID due to logistical reasons, which may have introduced confounding factors such as mental and physical fatigue, varying environmental conditions, or differences in participant motivation. However, it is important to note that pain levels during contraction did not differ between the HHD and ID measurements (see supplementary material 1), suggesting that pain exacerbation was not a significant factor. Randomising the order of measurements in future studies would help minimise the influence of these confounding factors.

Thirdly, the different testing postures required by the two instruments present another limitation. HHD assessments and ID measurements were performed in different positions, which may have influenced the measured trunk strength due to variations in muscle activation patterns, length-tension relationships, and biomechanical stabilisation demands. Despite this limitation, the testing position used with HHD has demonstrated validity and clinical utility, particularly in practical settings where portability and ease of use are priorities. These discrepancies underscore the importance of standardising testing positions to enhance the consistency and comparability of results across instruments while maintaining clinical applicability.

Finally, while the study included 21 participants, which may be considered modest for responsiveness studies based on COSMIN guidelines [14], the findings demonstrated moderate to large effect sizes for changes in trunk strength measured with the HHD, reflecting its internal responsiveness. Statistically significant differences were also observed in pain, disability, kinesiophobia, and health-related quality of life following the intervention. These results suggest that the sample size was adequate to detect meaningful changes in these outcomes and to explore the criterion validity of the HHD in this context. Nevertheless, larger studies are encouraged to validate these findings and further establish the responsiveness of HHD for assessing trunk strength in individuals with chronic low back pain.

## Conclusion

Following a six-week progressive resistance trunk exercise programme, the HHD demonstrated an adequate level of internal responsiveness for detecting changes in trunk muscle strength in people with chronic LBP. However, although the HHD shows promise in terms of detecting muscle strength over time, which is important clinically, the HHD lacks external responsiveness. This warrants further investigation.

## Supplementary Information

Supplementary Material 1.

## Data Availability

All data generated or analysed during this study are included in this published article [and its supplementary information files].

## Abbreviations

LBP: Low back pain
PBOMs: Performance‐based outcome measures
ID: Isokinetic dynamometer
HHD: Hand-held dynamometer
N: Newtons
N/m: Newtons/meter
Flex: Flexion
Ext: Extension
InnRot: Inner rotation
OutRot: Outer rotation
COSMIN: COnsensus-based Standards for the Selection of Health Measurement INstruments
PROMs: Patient-reported outcome measures
NPRS: Numeric pain rating scale
ODI: Oswestry Disability Index
SF-36: 36-Item Short Form Survey
IPAQ: International Physical Activity Questionnaire
TSK: Tampa Scale for Kinesiophobia
MVIC: Maximum voluntary isometric contraction
ICC: Intraclass Correlation Coefficients
ES: Effect size
SD: Standard Deviation
AUC: Area Under the ROC Curve
SRMs: Standardised response means
PILE: Progressive isoinertial lifting evaluation test
