# Supplementary material for: Responsiveness of hand-held dynamometry for measuring changes in trunk muscle strength in people with chronic low back pain
Source: BMC Musculoskelet Disord. 2025 Jan 18;26:66. doi: 10.1186/s12891-025-08325-4 (PMC11742789; doi:10.1186/s12891-025-08325-4)
Supplement: Supplementary file 1 — Supplementary Material 1. [file 12891_2025_8325_MOESM1_ESM.pdf]

Supplementary material (1)

Comparison of Contraction Pain Levels Between Hand-Held Dynamometry (HHD) and Isokinetic Dynamometry (ID): Results of Wilcoxon Signed-Rank Tests

Tests of Normality

	Kolmogorov-Smirnov[a]			Shapiro-Wilk		
	Statistic	df	Sig.	Statistic	df	Sig.
Pre_Flex_HHD	.221	21	.009	.860	21	.006
Pre_Flex_ID	.228	21	.006	.903	21	.039
Post_Flex_HHD	.300	21	<.001	.820	21	.001
Post_Flex_ID	.237	21	.003	.821	21	.001
Pre_Ext_HHD	.298	21	<.001	.824	21	.002
Pre_Ext_ID	.332	21	<.001	.833	21	.002
Post_Ext_HHD	.318	21	<.001	.818	21	.001
Post_Ext_ID	.324	21	<.001	.800	21	<.001

a. Lilliefors Significance Correction

Hypothesis Test Summary

	Null Hypothesis	Test	Sig.[a,b]	Decision
1	The median of differences between Pre_Flex_HHD and Pre_Flex_ID equals 0.	Related-Samples Wilcoxon Signed Rank Test	.206	Retain the null hypothesis.

a. The significance level is .050.

b. Asymptotic significance is displayed.

Hypothesis Test Summary

	Null Hypothesis	Test	Sig.[a,b]	Decision
1	The median of differences between Post_Flex_HHD and Post_Flex_ID equals 0.	Related-Samples Wilcoxon Signed Rank Test	.763	Retain the null hypothesis.

a. The significance level is .050.

b. Asymptotic significance is displayed.

Hypothesis Test Summary

	Null Hypothesis	Test	Sig.[a,b]	Decision
1	The median of differences between Pre_Ext_HHD and Pre_Ext_ID equals 0.	Related-Samples Wilcoxon Signed Rank Test	.059	Retain the null hypothesis.

a. The significance level is .050.

b. Asymptotic significance is displayed.

Hypothesis Test Summary

	Null Hypothesis	Test	Sig.[a,b]	Decision
1	The median of differences between Post_Ext_HHD and Post_Ext_ID equals 0.	Related-Samples Wilcoxon Signed Rank Test	.206	Retain the null hypothesis.

a. The significance level is .050.

b. Asymptotic significance is displayed.
